# Chronic diseases and multi-morbidity - a conceptual modification to the WHO ICCC model for countries in health transition

**DOI:** 10.1186/1471-2458-14-575

**Published:** 2014-06-09

**Authors:** Tolu Oni, Nuala McGrath, Rhonda BeLue, Paul Roderick, Stephen Colagiuri, Carl R May, Naomi S Levitt

**Affiliations:** 1 Clinical Infectious Disease Research Initiative, Institute of Infectious Diseases and Molecular Medicine, University of Cape Town, Cape Town, South Africa; 2 Centre for Infectious Disease Epidemiology Research, School of Public Health, University of Cape Town, Anzio road, Observatory 7925, Cape Town, South Africa; 3 Academic Unit of Primary Care and Population Sciences, University of Southampton, Southampton, UK; 4 Africa Centre for Health and Population Studies, University of Kwazulu Natal, Durban, South Africa; 5 Department of Health Policy and Administration, Pennsylvania State University, State College, USA; 6 Boden Institute, University of Sydney, Sydney, Australia; 7 Faculty of Health Sciences, University of Southampton, Southampton, UK; 8 Division of Endocrinology and Diabetes, Department of Medicine, University of Cape Town, South Africa and Chronic Diseases Initiative for Africa, Cape Town, South Africa

**Keywords:** Chronic disease, Epidemiological transition, Multi-morbidity

## Abstract

**Background:**

The burden of non-communicable diseases is rising, particularly in low and middle-income countries undergoing rapid epidemiological transition. In sub-Saharan Africa, this is occurring against a background of infectious chronic disease epidemics, particularly HIV and tuberculosis. Consequently, multi-morbidity, the co-existence of more than one chronic condition in one person, is increasing; in particular multimorbidity due to comorbid non-communicable and infectious chronic diseases (CNCICD). Such complex multimorbidity is a major challenge to existing models of healthcare delivery and there is a need to ensure integrated care across disease pathways and across primary and secondary care.

**Discussion:**

The Innovative Care for Chronic Conditions (ICCC) Framework developed by the World Health Organization provides a health systems roadmap to meet the increasing needs of chronic disease care. This framework incorporates community, patient, healthcare and policy environment perspectives, and forms the cornerstone of South Africa’s primary health care re-engineering and strategic plan for chronic disease management integration. However, it does not significantly incorporate complexity associated with multimorbidity and CNCICD.

Using South Africa as a case study for a country in transition, we identify gaps in the ICCC framework at the micro-, meso-, and macro-levels. We apply the lens of CNCICD and propose modification of the ICCC and the South African Integrated Chronic Disease Management plan. Our framework incorporates the increased complexity of treating CNCICD patients, and highlights the importance of biomedicine (biological interaction). We highlight the patient perspective using a patient experience model that proposes that treatment adherence, healthcare utilization, and health outcomes are influenced by the relationship between the workload that is delegated to patients by healthcare providers, and patients’ capacity to meet the demands of this workload. We link these issues to provider perspectives that interact with healthcare delivery and utilization.

**Summary:**

Our proposed modification to the ICCC Framework makes clear that healthcare systems must work to make sense of the complex collision between biological phenomena, clinical interpretation, beliefs and behaviours that follow from these. We emphasize the integration of these issues with the socio-economic environment to address issues of complexity, access and equity in the integrated management of chronic diseases previously considered in isolation.

## Background

### Health transition and multi-morbidity

Many low and middle-income countries (LMIC) are undergoing rapid epidemiological transition with a rising burden of non-communicable diseases (NCD) [1-6]. In sub-Saharan Africa, this is occurring against a background of existing infectious chronic disease (ICD) epidemics, including HIV and tuberculosis (TB) [7,8], which has led authors to coin the term colliding epidemics of NCD and ICD [7]. This has been considered from an epidemiological and health systems perspective rather than the patient perspective. With HIV becoming a chronic disease associated with increased life expectancy, multi-morbidity, defined as the co-existence of more than one chronic condition in one person [9] is increasing. This has been shown to increase complexity in the management of these co-existing conditions [10] often resulting in poorer health outcomes, and higher health care needs and costs [11-13].

In high HIV-burden settings such as South Africa (SA), the premature ageing effect of HIV-infection [14], increased life expectancy, as well as the increased risk of dysglycaemia [15] and cardiometabolic disease [16] associated with some antiretrovirals, contribute to associative multiple morbidities and new disease constellations in the population. As a result of the growing problem of comorbid non-communicable and infectious chronic disease (CNCICD) in LMIC, a different pattern of multimorbidity to that described in high-income countries is emerging, and it is becoming evident at a younger age; the latter due to the younger age distribution of HIV-infected persons.

The Innovative Care for Chronic Conditions (ICCC) Framework developed by the World Health Organization provides a roadmap for health systems to meet the increasing needs of chronic disease care [17]. The model has at its centre, a triad of patient and family, community and health care team. The triad in turn is supported by the larger health care organisation, community and policy environment at the meso and macro level respectively. However, it does not significantly incorporate issues of multimorbidity, in particular CNCICD. This necessitates a re-thinking of models of healthcare delivery and health care policy and planning in order to provide an adequate primary and secondary care response to the ongoing health transition. We propose using the case study of South Africa, a country that is using the ICCC framework in its strategy to improve chronic disease management, to highlight the need to extend this framework to include the lens of multimorbidity and in particular CNCICD. We identify some gaps in the ICCC Framework at the micro, meso, and macro levels and propose inclusion of CNCICD complexity issues including disease interactions, factors that influence patient behaviour, health care provider choices and health system characteristics.

### Case study: South Africa

#### Burden of disease

The Global Burden of Disease study demonstrated that in Southern Africa, whilst HIV and TB rank 1st and 4th in the top ten causes of morbidity respectively, 50% of the causes of morbidity are NCD; cerebrovascular disease is ranked 7th and diabetes is ranked 8th [18]. In South Africa, morbidity and mortality from diseases of the circulatory system, neoplasms, endocrine, nutritional and metabolic disease are increasing [19]. Against this background, the burden of HIV and TB remain high with effective antiretroviral therapy (ART), in widespread use in South Africa since 2005/6. There are limited data on co-morbidities from South Africa. Unpublished data from a retrospective study on CNCICD using routine electronic databases from a public primary care clinic in a Cape Town informal township, found that 19% of HIV-infected patients on ART were receiving treatment for another chronic disease. Of these, 77% and 17% had co-existing hypertension and diabetes respectively (Oni, T et al; unpublished). The prevalence of hypertension and diabetes increased with age but was notably high in the younger age groups; at 20% and 12% in the 18-35 year olds rising to 30% and 26% in the 36-45 year olds respectively (Oni, T et al; unpublished). Another study in HIV-infected persons over 50 years old in rural South Africa reported that 39% and 51% of ART-experienced and naïve patients had a co-morbid chronic disease [20].

#### Health system

There are two significant reforms occurring within the South African public health system. Firstly, South Africa is developing a National Health Insurance (NHI) scheme, a financing system that aims to provide equitable essential healthcare to all. The focus in the first 5 years of implementation is on health system strengthening and a significant overhaul of the existing health system delivery structures will be required. Currently, a minority of the population receive health care through a private insurance system. The vast majority of citizens receive care through a public system, which is free at the primary care level, but is currently underfunded and overall offers significantly less access and fewer services. The proposed changes relate to infrastructure development, information management, and quality improvement with a focus on improving patient experience as well as ensuring accessible, equitable and cost effective care. Secondly, the National Department of Health is re-engineering primary health care to more effectively and equitably address the growing multiple disease burden. As part of this reform, NCD management has been prioritized and a comprehensive strategic plan to combat NCD proposed. Embedded in this strategic plan is the need to integrate all chronic disease management where possible [21]. Presently, most chronic diseases are managed in separate disease-specific primary care clinics.

**Figure 1:**
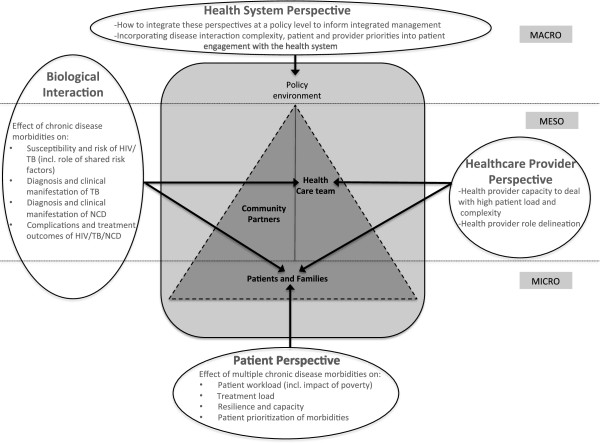
Conceptual modification of the WHO ICCC Framework: ICCC Framework represented within shaded square and modification represented by arrows and text bubbles.

This strategic plan was developed using the health systems building blocks described in the ICCC Framework [17]. The ICCC Framework identifies a need to shift from an acute episodic to a chronic model of care in developing countries. It speaks to integrated chronic care including coordination of care from prevention to tertiary care to improve the continuum of care. But since the inception of the framework in 2002, the epidemiology of population health has transitioned in the context of SA. The framework does not incorporate the concept of multimorbidity and particularly CNCICD. In addition, while it recognizes the central role of patients, family, and the community with an emphasis of the need for lifestyle and behavior change for effective management of chronic diseases, it does not incorporate the factors that influence these lifestyle choices and ability to effect behavior change and medication adherence. Several studies have proposed frameworks for exploring multimorbidity. These include examining the interaction between chronic diseases and the psychosocial environment [22], understanding patient workload [23] and incorporating knowledge of patient coping mechanisms and self-management skill requirements [24].We apply the lens of CNCICD and propose a framework that modifies the ICCC and the South African Integrated Chronic Disease Management (ICDM) plan to incorporate key components of relevance in countries like South Africa undergoing health transition. The proposed modified framework (Figure 1) incorporates micro-(e.g. individual patients and their interaction with the health care team), meso-(e.g. health care team and organisation), macro- (e.g. policies to integrate care) level factors that influence patient behavior, biological interactions between co-existing diseases and risk factors, contributing to increasing complexity; with implications on the health provider capability to manage these conditions and health system policies. We further use South Africa as a case study to demonstrate gaps identified by the application of our proposed ICCC Framework modification.

## Discussion

### Conceptual modification to the ICC framework

The conceptual modification builds on earlier clinical work that develops multimorbidity as a problem, and theoretical work [23,25] that proposes that patient workload and capacity interact to affect healthcare utilization and health outcomes. The proposed modification also emphasizes the importance of biomedicine (disease-disease interaction). It links this to patient and provider perspectives that interact with healthcare systems: it makes clear that healthcare systems must work to make sense of the collision between biological phenomena, their clinical interpretation, the beliefs and behaviours and their feedback on individual health outcomes,, and with the socio-economic environment to address issues of access, equity and cost effectiveness.

#### Biological interaction

Challenges associated with the management of multi-morbidity between chronic infectious, particularly HIV and TB and NCD have increasingly been reported in LMIC [7,26]. These multimorbidities may arise at different levels. At the individual level, multimorbidity sometimes cluster as a result of shared risk factors (e.g. obesity, diabetes and hypertension), or as complications of other diseases (e.g. chronic kidney disease leading to cardiovascular disease and vice versa). In addition, these previously distinct entities of NCD or ICD increasingly share the same upstream determinants of poverty and low socio-economic status [7]. CNCICD may interact with respect to clinical manifestation (studies suggest co-morbid type 2 diabetes may alter clinical manifestation of TB [27]), screening and diagnostic algorithms, susceptibility, and prognosis. In addition, challenges due to shared pathophysiology and toxicities, pharmacological interactions between treatments that may reduce efficacy or increase toxicity, and the bidirectional nature of some of these relationships (a long term sequelae of TB is chronic obstructive pulmonary disease (COPD); which itself increases the risk of TB), is important to understand [28]. These cases and their management are often more complex requiring more specialized, but paradoxically ‘minimally disruptive’ services [25]; challenging in under-resourced, over-stretched LMIC health systems.

In South Africa, most HIV and NCD patients are managed at the primary care level, primarily by nurses, with support from physicians as required. The colliding epidemics of HIV and NCD have highlighted the inadequacies of the existing vertical programs; and lend support to the call for a better-integrated health service. The WHO ICCC Framework recommends that health care workers work with community partners and patient households to improve health outcomes. Our proposed modification highlights the different levels of interaction to be considered with managing co-existing diseases. In this regard, research to better understand the population-specific epidemiology of the interactions between disease-specific morbidities, is required. The planned NHI in South Africa is an opportunity to integrate chronic HIV, TB and NCD care whilst ensuring coordination between chronic and acute services.

#### Patient perspective

The demographic characteristics of patients with multi morbidity and CNCICD in LMIC is such that there is a need to consider the ability of patients to self-manage and prioritise when they are economically active or have pivotal social responsibilities or roles in the household. Shippee et al [23] have set out a model of patient experience that proposes that treatment adherence, healthcare utilization, and health outcomes are influenced by the relationship between the workload that is delegated to patients by healthcare providers (including managing self-care, treatment modalities, behavior change, and clinic visits), and patients’ capacity to meet the demands of this workload (including physical and mental functioning, pre-existing health literacy and family and social support). In the Shippee model, multimorbidity is likely to both increase workload and reduce capacity – over time it adds complexity to patient experiences, and leads to ineffective and inappropriate outcomes. Notably, the burden of multimorbidity is generally higher in the poor who often have a lower capacity to deal with ill health. The workload/capacity balance and patient input into their overall self-care, contribute to patient prioritization and choices.

A key component of the South African NHI scheme is improving the quality of patient experience when accessing health care. We propose that consideration of the patient-centred experiences should include understanding factors that influence coping mechanisms, patient choices and prioritization when multiple morbidities co-exist. This calls for better context-specific measures of workload and capacity.

#### Health provider perspective

Analysis of professional-patient encounters in chronic disease management settings shows that patients consistently reveal intensification of self-care and self-management demands, and increasing workload with multiplication of comorbid conditions [29,30]. In this context, patterns of healthcare delivery that emphasize minimally disruptive healthcare [25] are likely to improve treatment adherence because they focus attention on understanding and engaging with patient preferences, improving capacity, and reducing workload. Consequently, management decisions are a process of trade-offs, dependent on the health provider’s ability to deal with a multiplicity of co-morbidities. Multimorbidity and CNCICD requires a shift towards integrated generalist care, rather than the continued proliferation of specialist roles, because it means that patients are no longer dependent on a single clinical discipline, but now need to interact with several. This increases the organizational and administrative workload placed on patients and adds problems of workability to that of the accumulation of complexity proposed by Shippee et al. [23]. The potential challenge of up skilling generalists could be countered through emphasis on integrated chronic disease teams. Therefore it is important that evidence from biological interactions and patient perspectives inform care of complex patients. A view of achievable health goals and prioritization of conditions, shared by the chronic disease team and patient, is desirable to enhance patient satisfaction, their adherence and respect for patient choices and clinical decisions alike.

#### Health system perspective

The current vertical chronic disease specific health system requires re-consideration in a setting of a high prevalence of multimorbidity. The South African NCD strategy comments on the need to integrate chronic infectious and non-communicable diseases although successful demonstration remains limited at this point. We propose extending the concept of integration to incorporate patient perspectives that take into account the complexity of social determinants of health and the capacity to respond to ill health. We also advocate the importance of incorporating the health provider perspective into informing the structuring of the health system including human capacity to improve management and outcomes of chronic diseases. One option for integration is in the community adherence clubs. Presently, separate HIV and NCD adherence clubs, coordinated by disease-specific community care workers (CCWs), exist where patients interact with other patients with similar chronic diseases, receive health education talks and are dispensed repeat prescriptions. Integration of training for CCWs and these clubs would facilitate continuity of care and easier access to routine prescriptions, and pilot studies of CCW integration are underway in South Africa. Another option is the integration of chronic disease clinics for patients considered to be stable. This would include integration of screening for other prevalent chronic diseases.

With respect to monitoring and evaluation, the Direct Observed Therapy (DOT) short-course framework for TB management has demonstrated that a standardized electronic information management system is crucial to facilitating effective monitoring and evaluation of the equity and effectiveness of chronic disease management systems and interventions. This includes, standardized indicators to measure single- and multi-morbidity. Pilot interventions to integrate chronic disease clinic stationary are underway in the Western Cape. The Western Cape province of South Africa conducts annual NCD audits to assess progress towards identified targets. This includes an audit of facility equipment, management, and individual disease management targets. Whilst lessons learnt from audits and the DOTs framework provide a starting point for examination of the health system model, these approaches do not sufficiently integrate other perspectives. Of note, the patient perspective, including the acceptability of interventions that take management of morbidities out the health facilities and into the home, should be incorporated into the monitoring and evaluation of the effectiveness and cost effectiveness of health system policy and interventions. In addition, the monitoring of equity in health services and the impact of multimorbidity and health system changes would be vital to reduce socioeconomic/geographical inequity gaps.

## Summary

The ICCC framework provides a comprehensive framework for integration of chronic disease management and is the cornerstone of primary health care re-engineering and the ICDM in South Africa. We argue that it is important to also take into consideration changing patterns of morbidity and the increasing prevalence of CNCICD, particularly in LMIC, using SA as a case study, where rapidly urbanizing poorer populations are arguably more susceptible to multimorbidity and the adverse outcomes that can result. Our proposed modification recommends incorporating biological interactions, patient and health provider workload and capacity into the ICCC Framework to inform primary heath care re-engineering and integrated chronic disease management to optimize health outcomes. In order to reach the target of a 25% reduction in mortality due to NCD by 2025 set at the 65th World Health Assembly, innovative strategies will be required. In South Africa, the ongoing health care reforms provide an opportunity to incorporate issues of complexity associated with multimorbidity and CNCICD into the strategic objectives of primary care re-engineering and integrated chronic disease management. Our proposed modification of the ICCC Framework could also be considered for dealing with CNCICD in other LMIC undergoing demographic, epidemiological and health transition.

### Ethics

There were no human subjects involved in the creation of this manuscript.

## Abbreviations

HIV: Human Immunodeficiency Virus; LMIC: low and middle-income countries; CNCICD: Comorbid non-communicable and infectious chronic disease; NCD: Non-communicable diseases; NHI: National Health Insurance; SANHANES: South African National Health and Nutritional Examination Survey; TB: Tuberculosis; ICCC: Innovative Care for Chronic Conditions Framework.

## Competing interests

The authors declare they have no competing interests.

## Authors’ contributions

TO was responsible for conceptualizing and drafting this manuscript. NMcG, RB, PR, SC, CRM and NSL contributed to the writing and editing of the manuscript. All authors read and approved the final manuscript.

## Pre-publication history

The pre-publication history for this paper can be accessed here:

http://www.biomedcentral.com/1471-2458/14/575/prepub
